# Normative Criteria and Their Inclusion in a Regulatory Framework for New Plant Varieties Derived From Genome Editing

**DOI:** 10.3389/fbioe.2018.00176

**Published:** 2018-12-19

**Authors:** David J. S. Hamburger

**Affiliations:** Faculty of Law, Chair of Constitutional and Administrative Law, Public International Law, European and International Economic Law, University of Passau, Passau, Germany

**Keywords:** genome editing, regulation, genetically modified organism (GMO), new breeding techniques (NBTs), CRISPR, genome edited plants, stakeholder interests

## Abstract

Any legal regulation has to take into account fundamental interests and concerns, whether of private or public nature. This applies in particular to the politically and socially sensitive question of regulating plant biotechnology. With the advent of new breeding techniques, such as genome editing, new challenges are arising for legislators around the world. However, in coping with them not only the technical particularities of the new breeding techniques must be taken into account but also the diverse and sometimes conflicting interests of the various stakeholders. In order to be able to draft a suitable regulatory regime for these new techniques, the different interests and concerns at play are identified. Subsequently, a determination is made on how these interests relate to each other, before regulatory concepts to reconcile the conflicting demands are presented. The examined normative criteria, which can have an impact on regulatory decisions regarding genome edited plants and products derived from them, include: industry interests, farmer interests, public opinion, consumer rights and interests, human health and food safety, food security, environmental protection, consistency, and coherence of the regulatory framework and ethical or religious convictions. Since those interests differ from country to country depending on the respective political, economic, and social circumstances, the respective legislator has the task of identifying these normative criteria and must find a suitable balance between them. To this end, a concept is developed on how the different interests can be related to each other and how to deal with conflicting and irreconcilable demands. Additionally, a legislator may have recourse to a number of further analyzed regulatory measures. An approval or notification procedure can be used for a risk assessment or a socio-economic evaluation. Coexistence measures and labeling provisions are able to reconcile interests that are at odds with each other and the precautionary principle can justify certain safeguard measures. As a result, the individual country-specific regulatory outcomes regarding genome edited plants are likely to be as manifold as the interests and regulatory measures at hand.

## Introduction

A crucial function of the rulemaking process and its end result is the reconciliation of various interests. Only a rule that balances conflicting views and concerns is perceived as fair and just. The perceptibility of such an intrinsic fairness is a corner stone of many regulatory efforts, since the effectiveness of norms and regulations depends in part on their societal acceptance (Davis et al., 1978, p. 75; Allott, 1981, p. 229, 235). However, different concerns are not only taken into account by lawmakers to ensure a just legislation, but also to respond to external demands of their constituency. Especially the rulemaking process of democratic societies is exposed to external influences through lobbying, pressure groups or public opinion (Friedman, 1977, p. 59–60; Kau and Rubin, 1981, p. 141; Friedman, 1986, p.771).

This applies likewise to the highly controversial matter of regulating activities relating to genetically modified organisms. The hardly reconcilable positions of environmental activists and industry lobbyists often resulted in a burdensome legislative process or a de facto stalemate as witnessed in the European Union (Dederer, 2016b, p. 147–50; Davison and Ammann, 2017, p. 13–14). With the advent of new breeding techniques, the question how to regulate biotechnology in a prudent manner arises once again.

To be able to formulate a suitable regulatory regime for these new techniques, it is decisive to identify the various interests and concerns at hand. Subsequently, a determination must be made as to how these interests relate to each other, before regulatory concepts to reconcile the conflicting demands can be applied.

## Genome Editing and New Breeding Techniques

The development and adoption of high-yielded crop varieties, together with the use of agro-chemicals and new methods of cultivation in the 1960s marked the beginning of a new era in agriculture (Farmer, 1986, p. 175–76). Although not undisputed (Shiva, 1991; Tilman, 1998, p. 211–12), this so called “Green Revolution” led to a large increase in productivity, a decline in food prices and an improvement of human welfare in the following decades (Evenson and Gollin, 2003, p. 759–61; Kush, 2005, p. 1).

Against the backdrop of new agricultural challenges in the form of extreme weather events (droughts, floods, heavy rainfall, and storms), decreasing soil fertility, and increasing resistance formation in plant pests, there is an ever-growing call for a “Second Green Revolution” (Wollenweber et al., 2005, p. 337; Lynch, 2007, p. 493–95; Davies et al., 2011; McAllister et al., 2012, p. 1011).

The aim of this envisaged agricultural revolution is the development of plant varieties that are able to counter these adverse effects. With the advent of so-called new breeding technologies (NBTs), a solution to these problems seems now within reach.

NBTs is a collective term for different newly developed plant breeding techniques which allow a faster and more precise development of new plant varieties (Lusser et al., 2011, p. 23–27; European Food Safety Authority, 2012c, p. 6–12). These new methods have all in common that some kind of artificially induced genetic alteration is involved in the creation of a new crop variety.

The most promising of these techniques is the so-called genome editing with engineered site-directed nucleases (SDNs). This method makes it possible to target a specific position in a genome and change the DNA at that position precisely in the way intended. Together with an ever-growing understanding of genetics and a better knowledge of the genes that are responsible for expressing a certain trait, genome editing is a powerful tool for the development of new plant varieties. Four different types of engineered nucleases are currently available: meganucleases, zinc finger nucleases (ZFNs), transcription activator-like effector nucleases (TALENs), and the clustered regularly interspaced short palindromic repeats system (CRISPR/Cas). The newest and since 2012 (Peng et al., 2016, p. 1219) rapidly adopted representative of that subsection of NBTs is the CRISPR/Cas-method. Since it is even easier to handle, less expensive and has more potential than its predecessors, it is at the center of attention when it comes to new developments in plant biotechnology (Kole et al., 2015, p. 10; Travis, 2015, p. 1456; Kamthan et al., 2016, p. 1647–49; Georges and Ray, 2017, p. 2).

In contrast to traditional genetic engineering, genome editing is way faster, more cost efficient and precise which allows for new areas of application (Abdallah et al., 2015, p. 195–97; Osakabe and Osakabe, 2015, p. 395–97; Wolt et al., 2016, p. 511–12). However, these new possibilities are also associated with newly emerging and partly conflicting interests.

## Demand for a Regulatory Overhaul

Before discussing those interests that influence legislative action, it is necessary to clarify why new regulatory issues arise when it comes to genome editing.

The need for a new regulatory framework is usually justified by a comparison of plants derived from traditional genetic engineering or conventional mutagenesis techniques and those derived from genome editing. The traditional recombinant DNA (rDNA) technology makes it possible for a plant breeder to introduce genes from any living organism into a plant, irrespective of their sexual compatibility (Academy of Science of South Africa, 2017, p. 29). The gene is incorporated at a random position into the genome of the organism without any *ex ante* control over the effect this insertion may have. The result is a new transgenic plant variety, which could not have evolved naturally. Conventional mutagenesis via radiation or chemical mutagen causes random undirected mutations in the genome. This leads to a plant that does not cross species boundaries and, at least theoretically, could have evolved naturally as well. Genome editing, on the other hand, enables the plant breeder to cause site-specific genetic changes that are—like mutations caused by conventional mutagenesis—indistinguishable from naturally occurring alterations in plant DNA. Since these changes could occur in nature or via conventional mutagenesis as well, it is argued that such genetic changes should be subject to a different regulation than transgenic plants.

The difference between traditional genetic engineering and genome editing is, however, not as clear-cut as it seems at first glance. More precisely, the genome editing technique can be used to cause mutations (small insertion or deletion), gene replacement, gene insertion, and site-directed deletions, or inversions (Curtin et al., 2012, p. 42–44). Regarding genome editing using SDNs, a distinction is made between three application methods (European Food Safety Authority, 2012b; Lusser et al., 2012, p. 232; Sprink et al., 2016, p. 1497; Wolt et al., 2016, p. 514; Voigt and Klima, 2017, p. 321): SDN-1, SDN-2, and SDN-3. SDN-1 applications cause a double-strand break without the addition of a repair template. Consequently, the break is repaired solely by the plant's own repair mechanism resulting in a mutation. In the case of SDN-2, a small repair-DNA-template is introduced together with the nuclease to create a site-specific predefined mutation. The cell's repair mechanism uses that template to repair the double-strand break by copying the genetic information of the template into the plant cell. The result is a mutation at the locus of the double-strand break in accordance with the provided template. SDN-3 is used to insert new genetic material into the plant cell. To this end, apart from the double-strand break a larger stretch of donor DNA is introduced into the cell and the plant's natural repair mechanisms incorporates the donor DNA at the locus of the double-strand break.

While plants derived from SDN-1 and SDN-2 are indistinguishable from their conventionally bred counterparts (Lusser et al., 2011, p. 69, 2012, p. 237; Schenkel and Leggewie, 2015, p. 265; Sprink et al., 2016, p. 1497; Townson, 2017, p. 11; Voigt and Klima, 2017, p. 321), SDN-3 can lead to transgenic plants, depending on its specific nature of application. At the same time, the techniques of traditional genetic engineering can also be used for the development of plants which do not cross species boundaries (i.e., cisgenesis) (Holme et al., 2013, p. 395–97; Ribarits et al., 2014, p. 184; Jogdand et al., 2017, p. 691–92). Therefore, the difference between traditional genetic engineering and genome editing is not that the one method creates transgenic plants while the other leads to non-transgenic varieties. Genome editing differs from traditional genetic engineering techniques mainly in its more precise, targeted and less burdensome application and its ability to overcome some of the limitations of traditional genetic engineering (Kamthan et al., 2016, p. 1647–49).

Consequently, the question of whether non-transgenic plants should be excluded from the strict regulation of genetic engineering existed already before the advent of genome editing (Schouten et al., 2006; Conner et al., 2007, 351; Rommens et al., 2007, p. 402; Jacobsen and Schouten, 2008; Waltz, 2011, p. 677; European Food Safety Authority, 2012a). Therefore, genome editing does not only raise exclusively new regulatory questions, but is also used to put regulatory issues, which have existed before, on the agenda again.

This view is confirmed by the fact that the term NBTs seems to be used in some cases to avoid expressions like genetic engineering or genetically modified. The wording “new breeding techniques” gives the impression that it describes methods sui generis with completely distinct regulatory demands. In this way, the pressure on the legislature to take action can be increased without being associated directly with the controversial matter of genetic engineering.

Notwithstanding the fact that the regulatory questions are not entirely new, compared to traditional genetic engineering and conventional mutagenesis, genome editing has special characteristics, which must be considered.

Due to the possibility of specifically targeting a certain gene sequence, unwanted side effects are far less likely. Genome editing may cause so called off-target effects but it is still more precise than the random insertion of genes by traditional genetic engineering (Vogel, 2012, p. 60) and causes far less unwanted changes than conventional mutagenesis (Kahrmann et al., 2017, p. 177). Additionally, over the past years researchers have managed to limit off-target effects associated with CRISPR/Cas9 (Cho et al., 2014, p. 137–38; Peng et al., 2016, p. 1227) or are able to use the underlying mechanism to target multiple sites at once (Hyams et al., 2018, p. 2184).

Moreover, genome editing is frequently only used for minor changes in the genome instead of the insertion of large DNA segments or the generation of numerous random mutations. These factors can have an impact not only on the risk assessment, but also on the applicability of the existing regulations. Therefore, legislators worldwide are asked to take those special attributes of genome editing techniques into account and to give them a suitable legal framework.

## Normative Criteria

However, a legislative effort will most likely take into account not only the technical specifics of genome editing, but also the various interests at hand.

This includes (1) industry interests, (2) farmer interests, (3) public opinion, (4) consumer rights and interests, (5) human health and food safety, (6) food security, (7) environmental protection, (8) consistency and coherence of the regulatory framework, and (9) ethical and religious convictions.

The following analysis has the purpose to show how these interests are able to affect legislation in manifold and substantive ways and in what way they assume the status of normative criteria for the legislative undertaking of regulating plants derived from genome editing.

### Industry Interests

#### Biotech Industry

Due to lobbying, economic considerations, and political self-interest, national legislation is usually prone to take into account the demands of the domestic industry. Therefore, the kind of expectations companies invested in biotechnology bear, can have a considerable effect on regulatory decisions.

##### Legislative and political support for marketing

Historically this interdependency between industry interest and political action can be witnessed by comparing the approach to genetic engineering in the USA and the EU since the 1970s. The far more extensive public spending on life science in the US compared to the EU encouraged the development of an innovative biotechnology sector in the US. At the same time, stricter rules on the use of pesticides were imposed in the EU and the US since the 1970s. While European companies tried to keep their competitive edge in agrochemicals by developing environmentally friendlier pesticides, the US biotechnology firms tried to meet the higher regulatory requirements by developing new plant varieties (Graff and Zilberman, 2007, p. 245). As a result, American companies have been engaged in biotechnology research from early on and therefore have dominated the development and commercialization of agricultural biotechnology since the beginning (Pan, 2002, p. 230; Owen, 2017, p. 19). European companies, however, whose focus was still on traditional agrochemicals, increasingly fell behind in this area.

As a consequence, European agrochemical companies potentially had an interest in slowing down the adoption of biotechnology, while their American competitors were trying to facilitate its breakthrough (Graff and Zilberman, 2007, p. 245–56; Zilberman et al., 2013, p. 202–03; Graff et al., 2015, p. 681–82). Since the political influence of industry stakeholders is the strongest in their respective home countries, the American biotechnology companies were able to influence the US legislation in their favor while a negative stance was able to become solidified in Europe (Graff and Zilberman, 2004, p. 2–3; Zilberman et al., 2013, p. 206). However, it would be an oversimplification to attribute the EU's (in)action to the lack of industry intervention only. In holding back the adoption of genetic engineering, the EU adopted an effective strategy to protect the competitiveness of the domestic agrochemical sector. In this way, the US biotechnology companies were not only blocked from access to the European market, but also the global adoption of biotechnology was slowed down considerably. Since genetically-modified agricultural products can only be imported into the EU if they have been subject to the approval procedure, the EU's de facto moratorium on GMOs has resulted in a restrained use of genetically modified plants in exporting countries (Pollack and Shaffer, 2009, p. 296; Laursen, 2013, p. 579; Adenle et al., 2017, p. 249–50).

As a consequence, the legislative attitude toward the adoption of NBTs will depend significantly on how much skin in the game the respective domestic industry has. At this early stage of research and development, it is difficult to make reliable predictions in that regard. However, there are first indications that the commitment of the scientific community and the biotech-industry is not as one-sided as it used to be concerning traditional genetic engineering. Figures from the year 2010 (Lusser et al., 2012, p. 233) show that 44% of the publications on NBTs were published by researchers from the EU whereas only 32% could be assigned to North America. This could lead to a shift in European policy toward a more embracing attitude when it comes to genome editing.

##### Protection of intellectual property rights

To work profitably, biotech companies must generate a steady revenue stream by selling their genetically modified plants. To prevent farmers from paying only once for the seeds by reusing their last crop, developers depend on the protection of their new plant varieties by intellectual property laws or a similar protective mechanism.

The likelihood of lawmakers accepting new plant varieties as intellectual property or protecting it in a comparable way depends mainly on the economic interest the respective country has in having access to such biotech products. As a result of past experience (Bronstein, 2016; Monsanto, 2016; Reuters, 2016), it is to be expected that producers will withhold new products from national markets as long as their effective protection is not ensured. Therefore, as long as the dependency of the domestic agricultural sector is significant enough, the biotech industry will be able to shape the regulatory framework in their interest.

In addition to the mere existence of an effective protection mechanism, the biotech industry needs to be able to determine if, where and by whom its products are used to collect the royalties. Since it is possible to create plants by means of genome editing which are indistinguishable of naturally mutated plants there are additional obstacles to the proof of origin.

This endeavor is less burdensome in legal orders that allow for a prima facie evidence. Even though it is possible that exactly the same mutation caused by genome editing also occurs naturally, it is, however, highly unlikely and utterly implausible on a large-scale. In that case, the farmers would bear the burden of proof and would have to show that the genetic alteration in their harvest originated from a natural process—an evidence that can de facto not be provided.

If such a prima facie evidence is not allowed and lawmakers cannot be pressed to adopt an amendment in that regard, an identity preservation system (IPS) could serve the industry interest as well. Since this would coincide with the interest of organic farmers, an IPS could turn out as a mutually agreeable solution.

Whether an IPS is actually in the interest of biotech producers, depends, however, on who bears the costs and how GM contamination is treated under national legislation.

In Germany, for example, GM farmers have to compensate their conventional or organic counterparts if a contamination of their harvest with GMOs makes it illegal to place their products on the market, requires to label the products as containing GM, or prevents them from using a certain label (e.g., “GM-free”) (Kohler, 2005, p. 566; Dederer, 2007, 2016a p. 222, 121). In that case, the biotech industry might have a certain interest in the existence of an IPS since otherwise the liability risk is likely to deter farmers from adopting GM technology. However, if biotech farmers have to bear the costs of an IPS alone, the deterrent effect would be mostly the same.

##### Streamlined approval or notification procedure

The costs caused by regulatory requirements or delays (Kalaitzandonakes et al., 2007, p. 509–10; Smyth et al., 2016, p. 185–87) can have a detrimental effect on the company profit and discourages new investments in the development of biotechnology innovations. However, it should be noted that an effective approval procedure is not just a private but a public interest as well. On the one hand, a time-saving procedure attracts investment in the domestic economy. On the other hand, public sector institutions are also engaged in the development of new crop varieties, especially in developing countries (Cohen, 2005, p. 32). Since they depend on public funding, high approval costs might cripple their efforts to provide a public good (Smyth et al., 2016, p. 188).

Therefore, it stands to reason that the biotech industry would welcome it if genome-edited plants fell outside the scope of a strict approval or notification procedure. However, the current market leaders might have a strong self-interest in a costly and burdensome approval procedure. This shields their market share from new competition and discourages smaller but innovative competitors to invest in research and development (Miller, 1997, p. 184). At the same time, the big biotech-companies have the sufficient cash flow, human resources, and past experience to work the system.

In any case, a streamlined regulatory framework has to be balanced against public safety and environmental issues (see below).

#### Organic Food Industry

The organic food industry not only positions itself as an environmentally friendly alternative to genetic engineering, but also actively combats the adoption of genetically modified plants (Apel, 2010, p. 636). From a purely economic point of view, that approach seems rather non-sensical since the delimitation to genetic engineering and conventional agriculture is an important selling point of organic farming. The abolition of GM plants would deprive organic farming of one of its most prominent distinguishing features.

The lobbying against genetic engineering can be partly explained as an expression of an agricultural idealism and the deeply rooted conviction that tempering with nature is inherently harmful.

However, it should not be left ignored that the opposition to GM techniques is also a very effective—though possibly unintended—marketing strategy. By establishing genetic engineering as an unmanageable risk to human health and the environment, a moral incentive to buy organic products is created. This is reinforced by consumers' fear of the negative consequences of the consumption of GM products. At the same time, the biotech industry as a common enemy serves as a catalyst to create a social movement with the aim to change the future of agriculture. The organic food industry managed to be recognized as the spearhead of that movement providing everyone with the opportunity to rally behind its cause. This way the production and consumption of organic food is not a mere economic process but also part of a political agenda.

In addition to this political motivation, the organic food industry has also a purely economic interest in hampering the widespread adoption of GM plants. The industry relies on the price premium consumers are willing to pay for organic food. A large-scale use of GM varieties would most likely lead to falling prices for non-organic agricultural products (Moschini et al., 2000, p. 48; Qaim and Traxler, 2005, p. 82; Brookes et al., 2010, p. 31–32). As a consequence, the gap between organic and non-organic products would widen. Surveys indicate, however, that consumers are willing to buy GM products when they are offered a significant discount (Lusk et al., 2005, p. 40; Knight et al., 2007, p. 508; Aerni et al., 2011, p. 835). A larger gap between consumer prices of organic and non-organic agricultural products could therefore significantly affect the market share of the organic food industry in a negative manner.

On the basis of these economic and political interests, it can be assumed that the organic food industry will take a negative stand in respect of genome editing and actively lobbying for a strict regulatory framework. The predominantly condemning policy statements regarding genome editing by non-governmental environmental organizations (GMWatch, 2014; Greenpeace, 2015; IFOAM-Organics International eV, 2015; GM. Freeze, 2016; Paul et al., 2017) are the first indicator for this development.

The impact those efforts will have on the law-making process will most likely depend on the degree of correlation between the interest of the organic food industry and public opinion or in other words on the level of correlation which can be suggested to policy-makers. Since legislators have an incentive to act in accordance with the opinion of their constituency (Denzau and Munger, 1986, p. 102), it can be assumed that interest groups are most effective when their policy aim is consistent with public opinion. However, to benefit from this nexus, it should be sufficient for interest groups to make the legislators believe that such a correlation exists.

### Farmer Interests

If the new breeding technologies can live up to their promise to increase yield while reducing the nutritional and climatic demands of plants, from the farmers' point of view, everything suggests a large-scale application of the new plant varieties.

This assumption is backed historically by the adoption of the previous generations of genetically engineered plants. Due to the increase in yield, the declined expenses for pesticides and the time-saving manner of application, the farmer's profit increased significantly—even if higher seed prices are taken into account (Qaim, 2009, p. 672; Smale et al., 2009, p. 11–32; Areal et al., 2013, p. 18–27; Carpenter, 2013, p. 251; Brookes and Barfoot, 2016). At the same time, a delayed adoption caused significant foregone income benefits (Kalaitzandonakes et al., 2016, p. 228 with further references).

There are still critical voices that doubt the economic value of genetically modified plants in agriculture (Greenpeace, 2008; Friends of the Earth, 2018). Those critics, however, find it difficult to explain why in countries, where farmers have the free choice between conventional and GM varieties, the adoption rate of the latter supersedes the former by a vast margin (Lucht, 2015, p. 4255). This contradiction could be explained only with the unreasonable assumption that the farmers are fundamentally inclined to act against their own economic interests.

External factors, on the other hand, can undermine those positive economic effects of genome edited new plant varieties.

The premium “GM-free” products are able to obtain in some national markets (Goodwin et al., 2015, p. 25) distorts the economic performance of conventional and GM varieties to a certain degree. Therefore, the group of farmers that benefits from this price premium has an incentive to refrain from adoption of genetically modified plants from a purely economic point of view. Since the farmers are only able to charge the premium if the unintended presence of GMOs can be prevented effectively, they have a strong interest that an identity preservation system is in place to assure the coexistence of GM and GM-free agriculture. To this end, a minimum distance between the different cultivation areas, separate processing facilities or a traceability system can be used to preserve the producers' freedom of choice. However, it must be noted that measures of coexistence result more often than not in a marginalization of genetically altered plant varieties since the rules securing coexistence are usually biased in favor of traditional agriculture. For example, due to possible liability risks, large distance space and a costly traceability system, GMOs are de facto prevented from having a significant share in acreage in Japan and the EU (before the cultivation was banned in many member states) (Varela, 2010, p. 353; Sato, 2015, p. 15–16).

Moreover, not all farmers act solely out of economic interests. Farmers who are not only guided in their activities by economic considerations, but also by their ethical, political or environmental convictions might be inclined to refrain from the adoption of genetically modified plant varieties.

Besides that, (especially European) farmers have an additional incentive to oppose a permissive regulatory framework regarding genome-edited crops. Since a ban, moratorium, or mere regulatory obstacles have the effect of a non-tariff barrier to trade, it is a potent method to shield the domestic market from international competition (Grossman, 2010, p. 125; Graff et al., 2015, p. 682; Phillipson and Smyth, 2016, p. 204).

In case the national agroindustry depends heavily on the export of agricultural products, farmers must also take into account their sales opportunities with their trading partners. If the regulatory framework of their main trading partner does not allow the importation of a certain genetically modified crop variety, farmers have no interest in this plant variety.

Summing up, the degree of interest farmers have to adopt new genome edited plant varieties depends on the economic viability of cultivating such plants. However, the economic benefits cannot be determined solely by comparing the agricultural performance of conventional with genome edited plant varieties. The individual country-specific external factors and the personal convictions of the farmers must be considered as well to draw a convincing conclusion regarding the farmers' interests.

### Public Opinion

In order to assess the role public opinion plays when it comes to the formulation of a regulatory framework for genome editing, it is decisive to understand the effect and impact public opinion has on public policy in general.

While it is mainly undisputed that public opinion can have an impact on the legislative process (Monroe, 1983, p. 38–39; Page and Shapiro, 1983, p. 175 with further references; Block, 1987, p. 65; Korpi, 1989, p. 323; Hill and Hinton-Anderson, 1995, p. 924 with further references; Stimson et al., 1995, p. 544; Smith, 1999, p. 860; Dahl, 2006, p. 131–32; Domhoff, 2014, p. 130–31), it is, however, unclear how strong its influence can be.

On the one hand, this depends on the respective political system. It is fair to say that the responsiveness to public opinion is pronounced in democracies (Dahl, 1971, p. 1). Regular elections, freedom of expression and an independent press allows a more direct interaction between the public will and the policy-making process. However, this does not mean that other political systems are completely lacking in dependence on the people's will. Even though dictatorships or authoritarian regimes can take less consideration of the public opinion (Peden, 1984, p. 360), they are not completely independent of it (Mueller, 1999, p. 139; Ojieh, 2015, p. 46–47). This circumstance is based on the fact that a lack of support in the society can be substituted only to a certain degree by the use of compulsory powers.

On the other hand, the way in which public opinion is articulated has a tremendous effect on its level of efficiency. Concentrated minority interests tend to have greater political influence than dispersed majority interests (Olson, 2002, p. 36). This could lead, for example, to a marginalization of the public interest by a contradictory but concentrated industry interest. In such a case, however, it should not be easily assumed that politicians have a reasonable incentive to act against public opinion. It seems more likely that they are simply unaware of the disparity between common and industry interest (Lohmann, 1993, p. 320; Burstein, 2003, p. 31). This danger is, however, mitigated by the fact that there is an increasing number of lobby groups representing consumer interests in a concentrated manner.

Moreover, the issue salience plays a central role for the degree of governmental responsiveness (Haider-Markel, 1999, p. 120). Since issues with a high salience are more likely to be taken into account by the voters on election day (RePass, 1971, p. 400; Jones, 1994, p. 14; Bélanger and Meguid, 2008, p. 479; McGrane et al., 2013, p. 5), politicians are more receptive to the public opinion on those matters. Nevertheless, public opinion on issues with a low salience is unlikely to be ignored completely due to the possibility that the emphasis shifts in the future (Burstein, 2003, p. 30).

Since genome editing is a fairly new technology, a nuanced public opinion on it has not yet emerged. However, it seems to be questionable if there will ever be a public opinion that differentiates between genome editing and traditional genetic engineering. The differences between the various methods of genetic engineering are of such an academic and technical nature that a differentiation by the public cannot reasonably be expected. It is also not more promising to ask for the position on transgenic and non-transgenic genetic modification, as this does not distinguish traditional genetic engineering and genome editing (cf. above). Therefore, it seems safe to assume that the existing public opinion on genetic engineering is going to find its continuation in relation to genome editing (similar Ishii and Araki, 2016, p. 1508).

Since there are significant regional differences concerning the public attitude toward the adoption of genetic engineering, the impact of public opinion on the regulation of genome editing has to be assessed by a country-specific case-by-case approach.

In general it can be stated that the public opinion on genetically modified food is more positive in developing countries than in the developed world (Li et al., 2002, p. 148; Curtis et al., 2004, p. 70; Pachico and Wolf, 2004, p. 159; Powell, 2013, p. 198 with further references). Looking closer at developed nations perceptions of GMOs are more favorable in North America than in Europe or Japan (Moon and Balasubramanian, 2001, p. 223; Lusk et al., 2005, p. 37; Lusk et al., 2006, p. 10; Vecchione et al., 2015, p. 330).

The existing surveys on consumers' attitude should be treated with caution, though. Due to the social stigma of GM products—especially in Europe—the adverse answers given in questionnaires can deviate significantly from the actual, more accepting behavior of consumers (Mather et al., 2011, p. 506; Desaint and Varbanova, 2013, p. 185; Sleenhoff and Osseweijer, 2013, p. 169–70).

In the end, the impact of public opinion on national legislation will depend mainly on the responsiveness of the political system, the issue salience, concentrated actions of like-minded interest groups and the lack of opposition from opposing societal or industry forces.

### Consumer Rights and Interests

When it comes to genetically altered products the interests and rights of the end-consumers also plays a significant role when tailoring a suitable regulatory framework.

One might assume that consumers have the right to have access to conventional and organic as well as to genetically modified food. However, it seems difficult to argue why there should be a legal right to have access to certain product categories. As long as there are no health concerns at play, this is rather a luxury than a necessity and therefore unlikely to be guaranteed by law. Nevertheless, even if there might be no right, there is certainly an interest of consumers in having access to organically or conventionally produced food next to genetically modified ones.

Additionally, there is a widespread assumption (Gruère et al., 2008, p. 1473)—sometimes even presented as fact—that consumers have the right to know if a product contains genetically modified material. A closer examination reveals, however, that while there is a consumer right to know in the EU [*Treaty on the Functioning of the European Union (TFEU)*, Art 169 (1)], other countries are much more reluctant to grant such a right with regard to labeling provisions (Keane, 2006, p. 292–93; Federici, 2010, p. 517).

However, even if there is no consumer right to information, a prevalent and substantial consumer interest in labeling might pressure legislatures to introduce corresponding laws. Against this backdrop, it can already be questioned whether the majority of consumers really wants to know if a product contains genetically altered material. Surveys show that consumers asked, if genetically modified ingredients should be labeled, are strongly in favor of such an obligation (The Mellman Group, 2012; Wunderlich and Gatto, 2015, p. 848; Committee on Genetically Engineered Crops Board on Agriculture Natural Resources Division on Earth Life Studies National Academies of Sciences Engineering Medicine, 2016, p. 303–04). However, the answers given to such a question should be treated with caution due to the inherent bias of that inquiry. When asked whether something should be labeled with regard to food, it is already implied that this information might be of significance for the consumer. It is also not plausible why a consumer would not want to know more about a product he is about to buy. It is therefore likely that a consumer will answer a question concerning the desire for further information in the affirmative, regardless of the content of that information. In a European consumer survey only 54.1% of respondents stated that they always read (or have previously read) the label before deciding to buy a particular food item (Sleenhoff and Osseweijer, 2013, p. 168). This is an indicator that consumers have a far lesser interest in proper food labeling in an actual shopping situation than anticipated. This is confirmed by the fact that consumers in countries, where a negative attitude toward genetically altered products prevails, are willing to buy genetically modified food products as long as they receive a price discount (Moses and Fischer, 2014, p. 67; Lucht, 2015, p. 4258–59). And even if consumers say that they do not buy genetically modified food, they often purchase them regardless (Sleenhoff and Osseweijer, 2013, p. 169). There is therefore a considerable discrepancy between the articulated and actually practiced interests of consumers with regard to the labeling of genetically modified products.

At the core of the interest of many consumers is, furthermore, the ability to purchase high quality products at low prices. The anticipated beneficial impact of genome editing on the nutritional value of food (Abdallah et al., 2015, p. 185; Khatodia et al., 2016, p. 9; Jiang et al., 2017; Karkute et al., 2017, p. 4; Lima et al., 2017, p. 238) combined with the expected market price drop (Voytas and Gao, 2014, p. 4–5; van Erp et al., 2015, p. 87) suggests that the adoption of products derived from genome edited plants would meet the consumer interest in that regard.

Additional indirect consumer interests may also result from considerations concerning health, food safety, food security, the environment, and ethical convictions (see below).

### Human Health and Food Safety

Decisive for the regulation of genome edited organisms (GEOs) are their implications for food safety and human health, since safety considerations are ordinarily the cornerstone of the regulatory efforts.

An assessment of these implications can be based on the potential toxicity, allergenicity, nutritional effects, and any unintended effects which could result from the genetic modification (World Health Organiziation, 2005, p. 12). It is, however, more often than not unclear which effects a GEO might have from an ex ante perspective. Therefore, an abstract regulation is only able to manage the general risk potential.

Against this backdrop, potential health risks of GEOs can be divided into four categories: the known knowns, the known unknowns, the unknown unknowns and the unknown knowns (For the origin of these general risk categories see U. S. Department of Defence, 2002; ŽiŽek, 2004; Daase and Kessler, 2016).

“Known knowns” means already clearly identified risks and certain knowledge of specific consequences of genome editing. This refers to such consequences that are already well-understood, like the fact that no different potential adverse effects can be attributed to plants bred via SDN-1/2 compared to plants resulting from conventional mutagenesis since the same genetic alterations can occur by means of both techniques. Furthermore, the lack of traceability/identifiability due to indistinguishability of certain GEOs from naturally occurring or conventionally induced genetic alterations can be mentioned in that context (Ribarits et al., 2014, p. 185–86).

“Known unknowns” describes the situation in which, although one is aware of the possibility of a risk, one does not know about the actual risk itself. This category includes, for example, off-target effects. In advance one does not know where they occur or what effect they might have, but it is clear that they can occur—even though off-target effects using genome editing are less likely compared to traditional techniques of genetic modification and conventional mutagenesis (cf. above). A further example would be the unauthorized use of genome editing with the help of so called “CRISPR home kits” (Sample, 2016) or an unauthorized form of application by using edited viruses and bacteria as biological weapon in a terrorist attack (Rodriguez, 2017, p. 4). Possible adverse long-term effects of artificial genetic modifications can be attributed to this category as well. With regard to genome editing the fairly unpredictable long-term effects of so called gene drives are just one example (Champer et al., 2016, p. 156–57; Chneiweiss et al., 2017, p. 712).

The “unknown unknowns” refer to those risks one does not even know if they exist. By nature of this risk category, it is not possible to give an example for such an unknown unknown. That is why it seems doubtful if an unknown unknown can be regulated at all—even if considering a maximal precautionary approach. With regard to GEOs a protection of unknown unknowns can only be guaranteed by refraining from the use of GEOs entirely. However, this could lead to the manifestation of a different unknown unknown arising from exactly that non-use of the technology. Consequently, an inclusion of unknown unknowns in a legislative effort seems not feasible.

The term “unknown knowns” applies to those risks that one is unaware of, although one actually knows or at least could know them. Since genetic modification is an extremely risk sensitive and risk aware area, an example for this category cannot be identified. It is worth considering, however, whether “unknown knowns” could be interpreted in a different way. Instead as suppressed risk, it seems more appropriate to read “unknown knowns” here as perceived risk even though its very existence has been scientifically disproven. This applies, for instance, to the often denied, but scientifically proved, lack of a specific risk inherent to genetic engineering as such (Dederer, 1998, p. 32–49).

Apart from the risk potential, GEOs can also have a beneficial impact on human health.

For instance, an improvement of the nutritional value of crops is frequently associated with genome editing (Abdallah et al., 2015, p. 185; Khatodia et al., 2016, p. 9; Jiang et al., 2017; Karkute et al., 2017, p. 4; Lima et al., 2017, p. 238). This is of special importance to developing countries since the population is often relying only on a single staple food—especially cereals—to meet their nutritional needs (Christou and Twyman, 2004, p. 35; Bouis, 2007, p. 79). However, the nutritional value of food is of lesser concern in countries where the population has access to a wide variety of food (Key et al., 2008, p. 292).

Positive effects on human health can also be the indirect result of beneficial impacts on food security and the environment (cf. section Food Security and Environmental Protection).

With regard to the legislative impact of effects on human health and food safety, it can be presumed that in developing countries the benefits are more likely to be considered as out-weighing potential risks, while developed countries might be more risk sensitive.

### Food Security

“Food security exists when all people, at all times, have physical and economic access to sufficient, safe and nutritious food to meet their dietary needs and food preferences for an active and healthy life” (World Food summit, 1996, Para.1). The global food security is increasingly under pressure due to an ever-growing world population (United Nations Department of Economic Social Affairs, 2017, p. 1), scarcity of arable land, the adverse effects of climate change (Mendelsohn and Dinar, 1999, p. 278; Olesen and Bindi, 2002, p. 246; Schmidhuber and Tubiello, 2007, p. 19703–04; Lobell et al., 2008), a higher per capita consumption (Godfray et al., 2010, p. 812), the vulnerability of monocultures (Altieri and Nicholls, 2004, p. 172; Georges and Ray, 2017, p. 5), and the formation of resistances in plant pests (Tabashnik, 1994, p. 47; Beckie, 2011, p. 1039; Tabashnik et al., 2013).

As a result of the population growth it is estimated that global agricultural production has to double until 2050 (Ray et al., 2013, p. 1). However, current rates of yield increase are not sufficient to meet this goal (Ray et al., 2013, p. 2). It is anticipated that the genome editing technique could close this gap due to its inexpensive, more precise, efficient and less time consuming nature of application (Ma et al., 2017). Against this backdrop, genome editing has shown promise for a more efficient disease control through a targeted mutation of specific disease-resistance genes (Georges and Ray, 2017, p. 5–6). With regard to climate change, it is expected that genome editing could lead to new cold, heat, or drought resistant crops varieties (Khatodia et al., 2016, p. 9; Scheben et al., 2016, p. 7). At the same time, genome editing can be used to increase the nutritional value of a plant product or knockout genes responsible for the production of anti-nutrients or allergens (Kamthan et al., 2016, p. 1649).

The presumed beneficial impact of genome edited crops on food security is more likely to lead to an embracing regulatory approach in those countries which already have to deal with malnutrition or are going to be adversely affected by climate change. Especially developing countries are often afflicted by both (Lobell et al., 2008), whereas Europe and the US might overall benefit from climate change from a purely agricultural perspective (Olesen and Bindi, 2002, p. 257; Reilly et al., 2003, p. 65). However, security interests regarding the countries affected by malnutrition and growing migratory pressure could also convince industrialized countries to rethink their attitude toward genetically modified crops. Since an agricultural surplus produced in industrialized countries would decrease the world market price, food, and feed would become more accessible to those struggling countries. This improved food supply could in turn lead to the desired stabilization and strengthening of destabilized regions.

### Environmental Protection

After decades of widespread environmental pollution and degradation, regulators and the public became more and more sensitive toward environmental protection issues. By now, environmental impact assessments and protective measures are a cornerstone of many regulatory endeavors. Any regulation of GEOs is therefore likely to include environmental considerations as well.

Potential risks for the environment include unintended effects on (non-)target organisms, the ecosystem, or biodiversity (Secretariat of the FAO/WHO Global Forum of Food Safety Regulators, 2005, p. 202). This includes among others off-target effects, the displacement of wild species by their stronger genome edited counterparts and unforeseen consequences of a gene-drive (Rodriguez, 2017, 2). The already established risk categories of known knowns, known unknowns, unknown unknowns and unknown knowns are here applicable as well.

However, the use of genome edited plants might also have a positive impact on the environment. As it has been observed in the case of GMOs (Smyth et al., 2015, p. 25–28), it seems reasonable to assume that the adoption of GEOs could result in less use of fertilizers and pesticides as well.

Furthermore, GEOs might have a positive effect on climate change. Higher yield gains of plants used for bioenergy production in combination with carbon capture and storage could increase the carbon removal rates (Humpenöder et al., 2014, p. 7). In addition, a higher yield could lead to less land use and make reforestation possible or could at least prevent further deforestation.

### Consistency and Coherence of the Regulatory Framework

In many societies the principle of the rule of law is deeply rooted in and a cornerstone of their legal system. The rule of law requires that laws comply with certain formal requirements: They should be general in nature, accessible by the public, prospective, coherent, consistent, compliable, and administered orderly (Fuller, 1969, p. 39; Raz, 1979, p. 214–18).

With regard to a regulatory framework for GEOs it is especially the consistency with other legal obligations and the coherence of the regulatory regime as such that might be at odds with the rule of law.

On the one hand, any domestic legislation must be in conformity with the applicable rules of international law. Against this backdrop, obligations originating from World Trade Law (Keane, 2006, p. 314–29; Kahrmann et al., 2017, p. 182) and Free Trade Agreements come to mind. It stands to reason that a different treatment of domestic conventionally bred plants and imported genome edited ones might clash with non-discrimination clauses.

On the other hand, the rule of law requires that the regulatory framework of GEO is coherent with other national laws by the same legislator. This raises the question of whether a different regulation of conventional mutagenesis and mutagenesis via genome editing is compatible with this principle. Since exactly the same outcome can be reproduced theoretically by either technique, it is rather difficult to argue why they should be regulated differently.

### Ethical and Religious Convictions

Ethical considerations are often referred to in order to oppose genetic modifications of plants. The main concerns articulated are (1) that humankind should not temper with the natural order (naturalness), (2) that the risk potential of genetic engineering cannot be estimated with sufficient certainty and its application is therefore unjustifiable (uncertainty), (3) the danger of corporate control over the food industry and exploitation of farmers via intellectual property rights, and (4) the failure to live up to the responsibility for further generations (Rollin, 2003, p. 15; Weale, 2010, p. 584–87; Rodriguez, 2017, p. 4).

The naturalness argument (1) is highly contentious (Rollin, 2003, p. 15–16; Weale, 2010, p. 584–85). There is no convincing logic argument why naturalness should be the benchmark for human action or why a natural state should be preferred ethically over an artificial one. Furthermore, it is often not possible to draw a sharp line between a natural and an artificial state (Weale, 2010, p. 584–85). Not even the crossing of species boundaries provides a clear demarcation line since this happens without human intervention as well (Weale, 2010, p. 585) and those boundaries are rather fluid (Rollin, 2003, p. 15; Robert and Baylis, 2005, p. 13–17).

With regard to the uncertainty of the risk potential (2) it seems at least questionable if uncertainty alone gives reason to an ethical imperative not to use genome editing at all. It seems to be more reasonable to demand that the technique is applied in a measured way.

The exploitation of the individual person by corporate or capital supremacy (3) is certainly contrary to generally accepted ethical values. However, agriculture is not more prone to be exposed to exploitation of the individual than any other industrial sector. The particularly pronounced fear of corporate control over the food chain can rather be qualified as an expression of an industry-skepticism instead of an actual ethical conviction.

Furthermore, it is argued that the responsibility for further generations (4) includes the obligation to leave behind a sufficient diversity of species (Rodriguez, 2017, p. 4). In that case, the application of a gene drive, which will eradicate an entire species, might be incompatible with this ethical demand. The same holds true for a release of such an invasive genome edited species that certain wild species become endangered.

On the other hand, there might even be an ethical imperative to use genome editing on plants. Since this is a promising method of combating malnutrition (cf. above), human suffering could be reduced significantly. Furthermore, a sufficient supply with agricultural products fosters peace and social justice within and among societies. It is anticipated that in the near future conflicts over increasingly scarce natural resources like water and arable land will intensify (United Nations Secretary-General Ban Ki-moon, 2007; Barnaby, 2009; Chellaney, 2013). A more equitable access of the world's population to agricultural products thanks to the adoption of GEOs might help to ease these tensions. A comparable argument can be made with regard to the anticipated mitigating effects of GEOs on climate change.

Religion, on the other hand, is often perceived as being in conflict with and slowing down scientific progress (Russell, 1997, p. 7). A more progressive approach, however, allows to assume that “there can never be a conflict between the broadening of scientific truth and the exercise of religious faith (…) [since] every new discovery reveals more about (…) God” (Grisham, 2012, p. 33) (similar Hathout, 1990, p. 99; Rispler-Chaim, 1998, p. 567; Ratanakul, 2010, p. 139).

From a Christian perspective humanity has a responsibility and a dominion of stewardship for God's creation (Grisham, 2012, p. 36). In a similar way the Qur'an prohibits to change God's creation [Haleem, 2005, p. 62 (4:119)]. Concerning the alteration of plants the mainstream of Islamic and Christian thought adopted the position that genetic engineering does not tamper with God's creation as long as it does not put it at risk and advances human welfare (Rispler-Chaim, 1998, p. 567; Fadel, 2001, p. 904; Conference of European Churches, 2001; Moosa, 2009, p. 142–46; Pope Francis, 2015, Para. 131). The Jewish tradition is even more permissive since it perceives humankind as co-creators with the task to complete God's creation (Green, 2010, p. 125). Therefore, a considerable number of Jewish scholars have no objections in general when it comes to the genetic engineering of animals and plants (Bleich, 2003, p. 67–71 with further references; Wolff, 2005, p. 924–25). Buddhists do not attach any unique or particular value to naturalness (Loy, 2009, p. 184) since they do not believe in a divine creator whose plan could be tempered with (Frazzetto, 2004, p. 554). They are therefore “not inclined to see a man-made creation as something competing with a “good” nature. There is a very positive attitude toward changing nature's course if it enhances the welfare of all living beings, and more so if it allows medical advancements” (Schlieter, 2004). Also in Hinduism there is no religious basis for an outright rejection of genetic modification *per se* (Narayanan, 2009, p. 175). On the contrary, Hindus are open-minded with regard to scientific advances and untroubled by the idea of tempering with a divine creation (Narayanan, 2009, p. 175–76). Even where genetically modified food could be in conflict with certain Hindu dietary rules, this can be neglected as long as there is a health benefit (Narayanan, 2009, p. 175).

In the end, the (mainstream) religious postulations are not at all that different from the already outlined secular factors: Human health, food security, and matters of environmental protection are to be taken into account by a regulatory framework for genome edited plants.

However, the existing opinions with respect to genetic engineering are in religious communities as diverse as in secular ones. Therefore, examples of strong religious opposition against genetic engineering of any kind can be found around the world (Epstein, 2001; Bleich, 2003, p. 67–68; World Council of Churches, 2005, p. 26–27; Moosa, 2009, p. 146–47; Omobowale et al., 2009, at Footnote 40; Loy, 2014, p. 268). As a consequence, in countries where a balanced position has no support and religious leaders have significant influence genetic engineering can face overwhelming obstacles.

It remains to clarify how those ethical and religious considerations can translate into law. Ethical and religious postulations can have a direct impact, if lawmakers are looking for external guidance when it comes to their own action. Religious stakeholders or pressure groups are able to influence lawmakers or public opinion by engaging in the discussion surrounding a legislative process and reaching out to their faith community. This is especially true for developing countries where a purely scientific point of view might be considered as threatening to longstanding traditions and customs (Omobowale et al., 2009, under the section “Discussion”). More often than not, however, ethical considerations are simply used to enforce an existing agenda by serving as an additional argument.

## Nexus of These Normative Criteria

At first glance, it stands to reason that the relation of the described different interests at play can be characterized as either corresponding, reconcilable or irreconcilable. However, the conducted analysis of the different categories of interests revealed that those are not homogeneous enough to make such a determination. For instance, with regard to environmental protection genome edited plants may have both beneficial and detrimental effects. The same holds true for human health considerations. The relationship between these two sets of interests alone is so complex and manifold that it cannot be narrowed down to the categories of “corresponding,” “reconcilable,” or “irreconcilable.” This is all the more true when trying to relate all the interests mentioned above with each other in a logically stringent manner.

Instead, a careful weighing and balancing of the different interests is far more promising. To this end, the significance, value and importance of each single normative criterion must be evaluated. As a result of this assessment, not all interest will turn out to be of such an importance that their inclusion in a legislative process is justified. This means that every criterion must meet a certain threshold of internal significance that makes it worth considering in the first place. The results of such an assessment will vary depending on the internal realities of the respective jurisdiction. For instance, the interest in food security is likely going to be less prominently featured in the regulatory approaches of industrialized countries, whereas public opinion might have a greater impact in democratic organized societies.

However, the criteria which have passed this threshold cannot all be treated alike.

There are the ones that are of such a high value that their weighing or balancing against other interests is not possible. Considerable health risks for a large number of people would fall into this category. However, in case that two or more interests of that kind are not completely aligned, an effort to achieve reconciliation by mutual effectiveness must be made. This can be achieved by finding such equilibrium between those that every single interest is able to unfold its maximally possible effectiveness under these circumstances.

On the other hand, there are also those criteria that are not absolute and therefore open for a weighing and balancing. This latter category of interests requires a clear assessment of their individual significance, before an appropriate weighing and balancing can take place. In case that an interest of that category is opposed to a normative criterion of absolute validity and it cannot be reconciled, the latter prevails.

**Figure 1:**
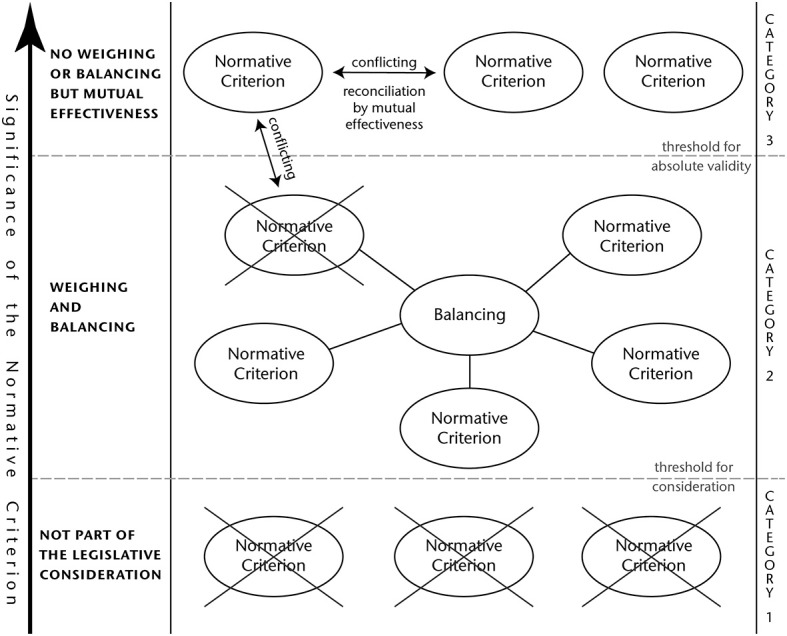
Nexus of the normative criteria and the regulatory implications.

A detailed visualization of this abstract concept can be found in Figure 1.

## Regulatory Concepts

The aforementioned abstract method to deal with the different normative criteria when considering a new regulatory framework must be embedded in regulatory concepts to make it applicable.

### Reconciling Regulatory Concepts

There exist several regulatory approaches that are designed to facilitate a weighing and balancing of different interests or to achieve at least a mutual effectiveness of conflicting normative criteria.

#### Approval or Notification Procedure

An approval or notification procedure before contained use, field trial, cultivation, or marketing of a GEO provides an opportunity to take into account the different normative factors mentioned above.

##### Risk assessment

A risk assessment can be used not just to determine possible adverse effects of GEOs but also to identify the importance those risks are going to have in a subsequent process of weighing and balancing.

Pursuant to the Codex Alimentarius (Codex Alimentarius Commission, 2003), a risk assessment of genetically modified food should include an investigation of direct health effects (toxicity), tendency to provoke allergic reactions (allergenicity), stability of the inserted gene, nutritional effects, and any unintended effects which could result from the gene insertion (World Health Organiziation, 2005, p. 12).

However, pursuant to the Codex those principles only apply to genetic modifications “that overcome natural physiological reproductive or recombinant barriers” (Codex Alimentarius Commission, 2003, Para.8). Therefore, those rules are not directly applicable to GEOs that were altered by means of SDN-1 and SDN-2 since they do not cross species boundaries. However, the Codex Alimentarius principles still provide a useful guidance regarding a risk assessment.

However, it should be noted that the more extensive a risk assessment is conducted, the more an approval is delayed and the more costly the market entrance and the final end product get. As a consequence, the desired scope of a risk assessment must be balanced and weighed against these interests, so that a risk assessment has to take place only to the necessary extent.

##### Socio-economic evaluation

The above-mentioned risk assessment is purely science-based without directly taking into account public opinion, ethical consideration, or societal values. It can therefore be argued that an approval procedure should allow such “soft” criteria to be included as well in the decision-making.

An example for this can be found in the Indian regulatory framework for GMOs which requires that a new genetic event is economically beneficial (Department of Biotechnology, 1998, Sec. 6; Pray and Bengali, 2005, p. 268–69). In the EU a new agricultural plant variety “must be of satisfactory value for cultivation and use” (Council of the European, Union, 2002, Art.4) to be allowed to enter the market. This is the case if “its qualities (…) offer (…) a clear improvement either for cultivation or as regards the uses which can be made of the crops or the products derived therefrom” (Council of the European, Union, 2002, Art.5 Para.4).

However, such an inclusion of non-scientific criteria raises concerns regarding its conformity with non-discrimination and anti-protectionism clauses of international trade regimes. Therefore, a case-by-case analysis has to determine if the introduction of a certain socio-economic approval requirement is legal in the first place.

#### Coexistence Measures and Identity Preservation Systems

Farmers have only a choice to cultivate either conventional, organic, or genetically modified crops if coexistence measures are adopted (European Commission, 2010, Sec.1.1). At the same time, consumers are only able to choose between conventional, organic or GM food if an identity preservation system allows for a proper labeling.

To protect such a freedom of choice, it must be prevented that the different product lines mix with each other. This could happen during cultivation by cross-pollination, through wind or bees, during harvest by contaminated equipment and during processing or transportation by (un)intentional mixture.

There is no uniform definition of “coexistence” and “identity preservation.” Consequently, the meaning of those terms varies significantly (Doshi and Lee, 2008, p. 305). Here, they are understood as concepts that build on each other but at the same time are distinct in nature.

The term “coexistence” is used hereinafter only for measures applied in the period from sowing to harvest and intended to ensure the coexistence of different plant organisms. Coexistence measures are, for instance, isolation distances or buffer zones between different crops, a required approval from neighboring farmers if minimum isolation distance is not respected, information duties (registration of areas in database, prior information to authorities, or neighbors), staggered sowing (different plant cycles and rotation intervals of sexually compatible GM and non-GM crops) and the cleaning/separation of equipment or obligatory insurances (Beckmann et al., 2014, p. 376; Lee, 2014, p. 244; Schenkelaars and Wesseler, 2016, p. 6–8).

An identity preservation system, as understood here, ensures that the segregation established by coexistence measures is maintained after the harvest until the product reaches the end-consumer. This is achieved, *inter alia*, with the help of an end-to-end paper trail, segregated production facilities, separate storage and testing procedures (Smyth et al., 2004, p. 140; Kumar and Sopory, 2008, p. 306; Wiseman, 2009, p. 257).

However, it should be borne in mind that coexistence and identity preservation measures can cause a de facto non-existence of genetically modified crops (Sato, 2015, p. 17). On the one hand, this is due to the fact that buffer zones cannot be maintained (Lee, 2014, p. 244) or the liability risk is too high. On the other hand, coexistence and identity preservation requirements increase the production cost (Falck-Zepeda, 2006, p. 1204; Gabriel and Menrad, 2015, p. 482, 484; Schenkelaars and Wesseler, 2016, p. 9). This might lead to a situation where the additional revenue from growing GMOs does not outweigh the extra cost due to coexistence measures (Venus et al., 2017, p. 421).

Another stumbling block for an identity preservation system with regard to GEOs is the fact that it is not possible to distinguish products derived from SDN-1/2 genome editing from naturally occurring mutations.

Here a distinction must be made between the terms “detection,” “identification,” and “traceability.” “Detection” refers only to the possibility to proof a certain genetic alteration. “Identification” means in this context that the origin of the detected genetic alteration can be verified (e.g., naturally or by means of a certain gene modifying technique). “Traceability,” on the other hand, stands for the capability to track GM-products at every stage of the supply chain by means of documentation and segregation (Ribarits et al., 2014, p. 185–86).

Keeping this in mind, the genetic alteration as such is detectable. However, at the moment it is not always possible to determine if that alteration occurred naturally or by means of genome editing. A detection of the origin of the genetic modification fails with respect to SDN-1, SDN-2 and certain forms of application of SDN-3 (Ribarits et al., 2014, p. 185–86; Eriksson, 2015, p. 35).

A monitoring of compliance and inspections would therefore be ineffective to some extent, if the competent authority has to prove the actual origin of the genetic alteration. However, this problem does not occur if the producer bears the burden of proof or a prima facie evidence is allowed, since it is implausible that a certain small, site-specific genetic alteration happened on a large scale naturally.

The coherence and consistency of such measures should receive special scrutiny with regard to GEOs as well. It could turn out to be difficult to argue why there should be measures in place to protect organic and conventional crops from GEOs if at the same time no measures are deemed necessary to protect organic farming from the non-organic methods of their conventional neighbors (e.g., a sprayed conventional crop protection agent also reaches the neighboring organic farmland). Concerning those GEOs that are indistinguishable from their conventional counterparts (SDN-1/2), a reasoning in favor of coexistence measures seems therefore to be difficult to uphold in a logically consistent manner.

#### Labeling

For consumers to have an actual choice between conventional, organic and GM food these products must be labeled. A prerequisite for labeling is the establishment of an identity preservation system as aforementioned.

However, the labeling of food containing material from GEOs faces several different obstacles. First of all, an end product which contains material created by means of SDN-1/2 is not physically different from products produced from a plant with the (theoretically possible) same genetic alteration but bred using conventional methods. A GEO label would therefore only inform about the manufacturing process, but not about the physical characteristics of the product. This makes the conformity of such a provision with WTO law at least questionable (WTO Panel Report, 1991, Para. 5.15; van den Bossche and Zdouc, 2017, p. 388–89).

It should also be borne in mind that labeling requirements cause additional costs (Kaye-Blake et al., 2004, p. 73; Federici, 2010, p. 556) and have a two-fold detrimental effect: On the one hand, they increase the selling price and thus reduce competitiveness. On the other hand, a labeling requirement for GEOs would imply that there is a well-founded reason to inform the consumer of that particular ingredient and might therefore act as a deterrent to the consumer in the same way as a warning notice would do.

Keeping in mind these adverse economic effects and the indistinguishability from conventionally breed plants, a mandatory labeling of GEOs might not be able to withstand a consistency or proportionality test.

#### Precautionary Principle

The precautionary principle as set out in Principle 15 of the Rio Declaration requires “[w]here there are threats of serious or irreversible damage, lack of full scientific certainty shall not be used as a reason for postponing cost-effective measures to prevent environmental degradation.” Even though the legal status of the precautionary principle as customary international law is still unsettled (Fitzmaurice, 2009, p. 4–6; Beyerlin and Marauhn, 2011, p. 284), it has been widely accepted (*Treaty on the Functioning of the European Union (TFEU)*, Art. 191 (2); Freestone, 1991, p. 36).

For the precautionary principle to be applicable there must (1) take place a scientific risk assessment (2) that identifies a potential but uncertain risk (3) whose realization would cause serious or irreversible damage (Andorno, 2004, p. 17–18).

The applicability of the precautionary principle to genome edited plants created by means of SDN-1/2 seems at least questionable. Since these plants are indistinguishable from natural ones, there can be no risk that goes beyond the natural “risk” of evolution. However, the precautionary principle is neither suitable nor meant to tame risks posed by nature.

With regard to the use of SDN-3, a case-by-case determination of the existence and gravity of a potential but uncertain risk should take place, since not every kind of application poses the same risk. Particular caution should be exercised to ensure that a mere hypothetical or perceived risk is not treated as a real but uncertain risk. With other words, the precautionary principle is suitable for the governance of known unknowns but not of hypothetical unknown unknowns.

However, “[t]he precautionary approach should not only consider possible risks, but also possible benefits and possible harms of a range of alternative options and their effect over people” (Rodriguez, 2017, p. 4). Therefore, the precautionary principle requires taking into account possible harms resulting from the non-use of genome editing as well. If those harms of non-use outweigh the risk of use to a certain extent, the actual use could be the “cost-effective measures to prevent environmental degradation.” Consequently, the precautionary principle could—under certain circumstances—also be used to justify the need to actually use the genome editing technique.

#### Opt-out

A viable option to mitigate such normative criteria that oppose an adoption of GEOs is to allow only certain types of usage and to opt-out of others.

This could mean, for example, that the import and sale of GEOs would still be allowed, but cultivation would be banned. Instead of a complete ban, a regional or geographically limited prohibition of cultivation is feasible as well, especially in federal states. In this way, areas that are ecologically particularly sensitive or where a negative public attitude toward genetically modified plants prevails could be exempted. This approach might appeal to a legislator in whose constituency the fear of release into the environment is particularly prevalent, widespread and pronounced.

If the opposition against GEOs is mainly based on the unwillingness to consume food that is derived from GEOs, it could be considered to prohibit the use of GEOs in food products but to allow the marketing of GEO feed instead.

If the public aversion to GEOs is caused by a perceived unnaturalness of genome editing, the legislator could restrict the use of SDN-3 while allowing SDN-1 and SDN-2.

If such restrictions are—as indicated here—not based on scientific grounds but rather on public opinion, political opportunism, or the pressure of interest groups, it might be difficult for advocates of genome editing to accept such constraints. However, it would be too short-sighted, to consider opt-out measures *a priori* as detrimental for the adoption of the genome editing technique. By partially giving in to the demands to regulate GEOs restrictively, the pressure and the mobilization potential to restrain the use of genome editing beyond that is reduced. This form of regulatory tradeoff can make the limited use of the genome editing technique possible in an otherwise rather unfavorable political or social environment. Therefore, a partial opt-out of certain types of application can actually be in the interest of GEO advocates as well.

#### Proportionality Test

The proportionality principle is enshrined in a wide variety of legal systems worldwide (Sweet and Mathews, 2008, p. 74–75, 112–60; Klatt and Meister, 2012, p. 1–3). It can therefore be assumed that a regulatory measure with regard to GEOs must at the same time comply with the principle of proportionality.

“The principle of proportionality requires that there be a reasonable relationship between a particular objective to be achieved and the means used to achieve that objective” (Clayton and Tomlinson, 2009, p. 323).

It is usually understood as consisting of four distinct parts (Rivers, 2006, p. 181; Craig and de Búrca, 2015, p. 551): (1) a legitimate objective must exist for the measure (legitimacy), (2) the measure must be suitable to achieve that objective (suitability), (3) the measure must not be more restrictive than necessary (necessity), and (4) the measure must not be excessive in relation to the objective pursued considering competing interests (balancing).

A measure's legitimacy is assumed if its purpose is lawful. Therefore, the pursuit of any of the normative criteria analyzed above should in general constitute a legitimate objective.

The suitability of the individual measure requires closer scrutiny. Even though a regulator is granted a certain margin of appreciation, the assumption of a measure's suitability must be based on factual grounds in order to prevent arbitrariness (Harbo, 2015, p. 72; Henckels, 2015, p. 53–54). Any measure addressing a non-existing risk is therefore *a priori* unsuitable. With regard to uncertain risks, a risk assessment can provide a factual basis for an envisaged measure. In case of a mere hypothetical risk, the permissibility depends on the scope of discretion that a legislator is granted by the applicable legal system.

The necessity test will most likely require a precise differentiation between SDN-1,-2, and−3, since it seems rather unlikely that it is necessary for a measure to encompass all the different genome editing methods in the same manner.

The last step of the proportionality test (balancing) is a suitable instrument to perform the weighing of category 2 normative criteria or to ensure the mutual effectiveness of conflicting category 3 criteria (cf. Figure 1).

Consequently, the proportionality doctrine serves the purpose to reconcile different normative criteria. As such, it is predestined to support the legislator when it comes to find a balance between the different interests existing with regard to the regulation of GEOs.

### Clear-Cut Regulatory Concepts: Ban or Non-regulation

In contrast to the methods mentioned above, which are based on balancing and reconciliation, clear-cut and one-sided approaches can also be considered regarding the regulation of GEOs. Such an approach could take shape in the form of a ban or even a non-regulation of GEOs.

A regulator might come to the conclusion that one or several normative criteria of absolute validity, which are not in conflict with opposing criteria of the same category (cf. Figure 1), make a complete ban of GEOs necessary.

This might be the case in societies where the slightest risk to the ecosystem weighs so heavily that a ban is perceived as the only regulatory option.

The opposite is also conceivable, namely that a regulation of GEOs is not deemed necessary or even that an unregulated status of GEOs is explicitly desired.

This scenario is feasible if possible adverse effects of GEOs do not pass the threshold for absolute validity or if the adoption of GEOs is backed by a normative criterion of absolute validity (cf. Figure 1). This might be the case in countries where GEOs are perceived as imperative solution to battle under- or malnutrition of the population.

However, both of these extreme scenarios are rather unlikely to be implemented in any jurisdiction. For a completely unregulated status of GEOs the issue of genetically modified organisms is by far too controversial. Against a complete ban speaks the fact that it seems difficult to put forward objective reasons to outlaw all forms of genome editing when keeping in mind the indistinguishability of SDN-1 and SDN-2 modifications from naturally occurring alterations.

## Conclusion

The analysis of normative criteria has shown that a regulatory framework for genome edited plants and products derived from them is influenced by a versatile accumulation of different interests.

Since those interests differ from country to country depending on the respective political, economic, and social circumstances, the respective legislator has the task of finding a suitable balance between these normative criteria. Although the interests are partly at odds with each other, regulatory tools are in place to reconcile most of them.

As a result, the individual regulatory outcome might be as manifold as the interests at hand, but should be within the restraint of international law and basic legal principles.

## Author Contributions

The author confirms being the sole contributor of this work and approved it for publication.

### Conflict of Interest Statement

The author declares that the research was conducted in the absence of any commercial or financial relationships that could be construed as a potential conflict of interest.
